# Bioactive Indole Derivatives from the South Pacific Marine Sponges *Rhopaloeides odorabile* and *Hyrtios* sp.

**DOI:** 10.3390/md9050879

**Published:** 2011-05-24

**Authors:** Arlette Longeon, Brent R. Copp, Elodie Quévrain, Mélanie Roué, Betty Kientz, Thierry Cresteil, Sylvain Petek, Cécile Debitus, Marie-Lise Bourguet-Kondracki

**Affiliations:** 1 Laboratoire Molécules de Communication et Adaptation des Micro-organismes, UMR 7245 MNHN-CNRS, Muséum National d’Histoire Naturelle, 57 rue Cuvier (C.P. 54), 75005 Paris, France; E-Mails: longeon@mnhn.fr (A.L.); quevrain@mnhn.fr (E.Q.); mroue@mnhn.fr (M.R.); kientz@mnhn.fr (B.K.); 2 Department of Chemistry, The University of Auckland, Private Bag 92019, Auckland, New Zealand; E-Mail: b.copp@auckland.ac.nz (B.R.C.); 3 Institut de Chimie des Substances Naturelles, CNRS UPR 2301, Centre de Recherche de Gif, avenue de la Terrasse, 91198 Gif sur Yvette Cedex, France; E-Mail: thierry.cresteil@icsn.cnrs-gif.fr (T.C.); 4 Centre Polynésien de Recherche sur la Biodiversité Insulaire, UMR 7138 CNRS, B.P. 529, 98713 Papeete, Tahiti, Polynésie française, France; E-Mails: sylvain.petek@ird.fr (S.P.); cecile.debitus@ird.fr (C.D.)

**Keywords:** indole derivatives, bromoindoles, marine sponge, *Rhopaloeides odorabile*, *Hyrtios* sp., PLA_2_ inhibitor, antioxidant, cytotoxic

## Abstract

Indole derivatives including bromoindoles have been isolated from the South Pacific marine sponges *Rhopaloeides odorabile* and *Hyrtios* sp. Their structures were established through analysis of mass spectra and 1D and 2D NMR spectroscopic data. Their potential inhibitory phospholipase A_2_ (PLA_2_), antioxidant and cytotoxic activities were evaluated. The new derivative 5,6-dibromo-l-hypaphorine (**9**) isolated from *Hyrtios* sp. revealed a weak bee venom PLA_2_ inhibition (IC_50_ 0.2 mM) and a significant antioxidant activity with an Oxygen Radical Absorbance Capacity (ORAC) value of 0.22. The sesquiterpene aureol (**4**), also isolated from *Hyrtios* sp., showed the most potent antioxidant activity with an ORAC value of 0.29.

## 1. Introduction

A great variety of simple and substituted indole derivatives, including halogenated indoles, bisindoles and tryptamine derivatives, have been previously isolated from marine organisms [1]. Indole derivatives are known to display various bioactivities such as anticancer, antibiotic, and anti-inflammatory activities [2]. Antioxidant activities were also recently reported for some analogues such as 2,2-diphenyl-1-picrylhydrazyl (DPPH) radical scavengers, highlighting an additional bioactivity in the series [3].

In our ongoing search for bioactive compounds within the frame of the CRISP program (Coral Reef Initiative in the South Pacific), the crude extracts of two South Pacific marine sponges were investigated, based on their significant anti-PLA_2_ activities. One specimen of *Rhopaloeides odorabile* was collected from the Solomon Islands and one specimen of *Hyrtios* sp. from the Fiji Islands. Fractionation of each of the crude extracts led to the isolation of a series of indole derivatives.

Three known monomeric indoles were isolated from the marine sponge *R. odorabile* and five dibromoindole derivatives, including the new derivative, 5,6-dibromo-l-hypaphorine (**9**), in addition to the sesquiterpene aureol (**4**) were obtained from the sponge *Hyrtios* sp.

The current report describes the isolation of alkaloids **1**–**9** and structural identification of the new analogue, 5,6-dibromo-l-hypaphorine (**9**). Anti-PLA_2_, antioxidant and cytotoxic activities of the series were evaluated and are presented.

## 2. Results and Discussion

### 2.1. Isolation of Indole Derivatives

**Figure 1.**
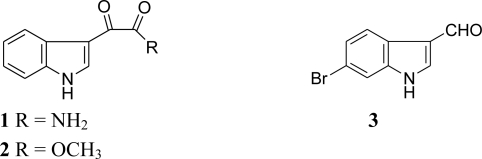
Structures of indole derivatives **1**–**3** isolated from the marine sponge *Rhopaloeides odorabile*

Successive chromatographic fractionation of the CH_2_Cl_2_ extract of *R. odorabile* using silica gel column chromatography and purification of the anti-PLA_2_ fractions on C18 HPLC afforded three known monoindole alkaloids (1*H*-indol-3-yl) oxoacetamide (**1**) and (1*H*-indol-3-yl) oxoacetic acid methyl ester (**2**), both previously isolated from the marine sponge *Spongosorites* sp. collected off the coast of Jeju Island, Korea [4] and 6-bromoindole-3-carbaldehyde (**3**) from the marine sponge *Pseudosuberites hyalinus* [5] (Figure 1).

**Figure 2.**
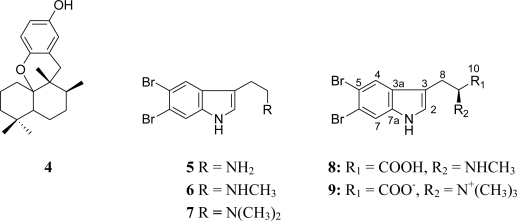
Structures of compounds **4**–**9** isolated from the marine sponge *Hyrtios* sp.

Chromatographic fractionation of the CH_2_Cl_2_ extract of *Hyrtios* sp. using silica gel afforded aureol (**4**), rapidly identified by comparison with literature data [6]. Chromatographic fractionation of the MeOH extract of *Hyrtios* sp. using C18 and LH 20 columns followed by successive ODS C18 HPLC revealed the presence of five dibromoalkaloids **5**–**9**. The structures of the known compounds **5–8** were rapidly determined as 5,6-dibromotryptamine **5**, *N*-methyl-5,6-dibromotryptamine (**6**) [7], *N*,*N*-dimethyl-5,6-dibromotryptamine (**7**) [8], and 5,6-dibromoabrine (**8**) [9] by comparison with literature data. The structure of the new metabolite, 5,6-dibromo-l-hypaphorine (**9**), was obtained through detailed examination of mass spectrometric data and extensive 1D and 2D NMR studies (Figure 2).

### 2.2. Structure Elucidation of 5,6-Dibromo-l-hypaphorine (**9**)

Compound **9** was isolated as an optically active pale yellow oil, with [α]^20^_D_ +28 (*c* 0.06, MeOH–1 N HCl, 8:2). The positive mode ESI mass spectrum of **9** showed a 1:2:1 molecular ion cluster at *m/z* 402.9, 404.9, 406.9, characteristic of the presence of two bromine atoms and corresponding to the molecular formula C_14_H_17_N_2_O_2_^79^Br_2_ for the pseudomolecular ion [M + H]^+^ at *m/z* 402.9667. ^1^H and ^13^C NMR data for **9** in DMSO-*d*_6_ were similar to those reported for 5,6-dibromoabrine (**8**), in particular the resonances of three singlet protons in the aromatic region at δ_H_ 8.02 (1H, s), 7.72 (1H, s), 7.27 (1H, s), a methine proton at δ_H_ 3.67 (1H, dd, *J* = 10.1, 3.3), methylene protons at δ_H_ 3.21 (2H, m) and the presence of a carboxylate function at δ_C_ 167.0 (C). The main difference between **8** and **9** was the presence of a N^+^Me_3_ cation, indicated by a nine-proton singlet in the ^1^H NMR spectrum of **9** at δ_H_ 3.17 (9H, s). In addition, two broad singlet protons at δ_H_ 11.20 (1H, brs) and 8.45 (1H, brs) suggested the presence of an amine and hydroxyl function, respectively. Furthermore, COSY correlations between the methine proton at δ_H_ 3.67 with methylene protons at δ_H_ 3.21 and between the amine proton at δ_H_ 11.20 with proton at δ_H_ 7.27 indicated the presence of a CH_2_-CH group and a NH-CH group, respectively. Five non-protonated aromatic carbons at δ_C_ 135.7 (C-7a), 128.2 (C-3a), 114.8 (C-6), 112.6 (C-5) and 109.5 (C-3) suggested 5,6 dibromosubstitution of the indole nucleus, which was supported by the observed HMBC correlations as presented in Table 1 and by comparison with literature values for **8** [9]. Thus, the new alkaloid, was identified as 5,6-dibromo-l-hypaphorine (**9**), a new member of the hypaphorine family. Halogenation on the benzene ring of tryptophan derivatives does not affect the sign of optical rotation [10], therefore **9** was assigned as l-configuration (9*S*) by comparison of its optical rotation value, [α]^20^_D_ +28 (*c* 0.06, MeOH–1 N HCl, 8:2), with those reported in the literature for 6-bromo-d ([α]^17^_D_–27 (*c* 0.8, MeOH, TFA, 8:1)) [11] and l-hypaphorine ([α]^15^_D_ +58 (*c* unspecified, MeOH, TFA, 8:1)) [12].

Several halogenated indoles bearing a *N*,*N*,*N*-trimethyltryptophane betaine moiety including di- and tri-iodo as well as both chlorine and iodine atoms on the indole nucleus have been reported from the Caribbean sponge *Plakortis simplex* [10,13]. The monobromoderivatives d-6- and l-6- bromohypaphorine were previously reported from the Okinawan marine sponge *Aplysina* sp. and from the sponge *Pachymatisma johnstoni*, respectively [11,12] and the dibromoderivative 5,7-dibromo-l-hypaphorine was previously obtained by synthesis [12]. This is the first report of 5,6-dibromo-l-hypaphorine (**9**) as a natural product.

### 2.3. Biological Activities of Compounds **1**–**9**

Compounds **1**–**9** were evaluated for their inhibitory activity against bee venom PLA_2_ and their antioxidant activity was estimated with the ORAC assay. The results from the *in vitro* assays are presented in Table 2. In addition, their cytotoxicity against the human pharyngeal carcinoma cell line was also determined.

Dibromoindoles **5**–**9** exhibited the strongest inhibitory activity against bee venom PLA_2_, with **9** being identified as the most efficient PLA_2_ inhibitor of the series albeit with a weak IC_50_ value of 0.2 mM.

**Figure 3.**
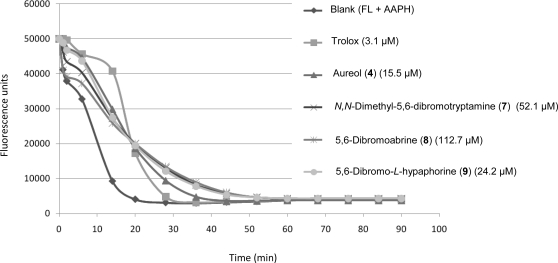
Fluorescein fluorescence decay curve induced by AAPH in the presence of Trolox or compounds **4**, **7**–**9**.

Compounds **4** and **7–9** displayed a positive antioxidant activity as first observed in the qualitative DPPH assay. Their antioxidant capacity was quantified in the ORAC assay measuring the loss of fluorescence of fluorescein (FL) in presence of the oxidative species AAPH (2,2′-azobis(2-amidinopropane dihydrochloride)) and using Trolox^®^ (6-hydroxy-2,5,7,8-tetramethylchroman-2-carboxylic acid), a water-soluble analogue of vitamin E, as an anti-oxidant standard against which all the compounds were compared. Compounds **4** and **9** exhibited the strongest antioxidant effect with a relative ORAC value of 0.29 and 0.22, respectively, as compared with Trolox which had value of 1. Figure 3 shows the FL fluorescent decay curves of the four derivatives (**4**, **7**–**9**) tested at different concentrations in order to obtain a profile similar to Trolox. Compounds **7** and **8** were the least effective antioxidants. Compound **9** was 4-fold less active than Trolox, displaying a similar curve at 24.2 μM, whilst compound **4** demonstrated a similar curve to Trolox at 15.5 μM, revealing it to be 3-fold less active than Trolox.

None of the isolated compounds demonstrated any cytotoxicity towards KB cells at 10^−4^ M except **4** (IC_50_ 5 μM), already known for its antitumor activity [6,9].

## 3. Experimental Section

### 3.1. General Experimental Procedures

Optical rotations were recorded on a Perkin Elmer 341 polarimeter. UV spectra were recorded on a UVIKON 930 spectrometer and IR spectra were recorded on a FT-IR Shimadzu 8400 S spectrometer. NMR spectra were obtained on a Bruker AVANCE 400 spectrometer. HSQC and HMBC experiments were acquired at 400.13 MHz using a ^1^H-^13^C Dual probehead. HMBC spectra were optimized for 7 Hz coupling. Mass spectra were recorded on an API Q-STAR PULSAR I of Applied Biosystem. HPLC were performed with an Alliance apparatus (model 2695, Waters) equipped with a photodiode array detector (model 2998, Waters), an evaporative light-scattering detector (model Sedex 80, Sedere) and the software Empower. HPLC solvents were purchased from Carlo-Erba.

### 3.2. Animal Material

Specimens of *Rhopaloeides odorabile* (class *Demospongiae*, order *Dictyoceratida*, family *Spongiidae*) and *Hyrtios* sp. (class *Demospongiae*, order *Dictyoceratida*, family *Thorectidae*) were collected from Solomon (8°58.968′S, 159°21.953′E) and Fiji islands (16°13.950′S, 179°01.900′E), respectively. Samples were identified by John Hooper (Queensland Museum, Brisbane). A voucher specimen is available for each under the accession numbers G322727 (*Rhopaloeides odorabile* R3101) and G324650 (*Hyrtios* sp. R3268).

### 3.3. Extraction and Isolation

Lyophilized sponge sample *Rhopaloeides odorabile* (20 g) was extracted with CH_2_Cl_2_ (5 × 200 mL, sonicated each time for 15 min) at room temperature. The five extracts were filtered, combined and concentrated under reduced pressure to yield 4 g of CH_2_Cl_2_ extract which was chromatographed on a silica gel (Merck) column using an initial gradient of cyclohexane/ethyl acetate from 80/20 to 60/40 followed by a second gradient of CH_2_Cl_2_/acetone from 80/20 to 60/40. The 80/20 CH_2_Cl_2_/acetone fraction (98 mg) exhibited anti-PLA_2_ activity and was submitted to semi-preparative reversed-phase HPLC column chromatography (Interchim, Uptisphere C18 7.8 × 250 mm) and eluted with increasing amounts of MeOH in H_2_O (flow rate: 3 mL/min, wavelength: 254 nm) through a linear gradient (10% to 100% of MeOH) for 35 min and afforded three compounds **1**–**3** (**1**: 20 min MeOH/H_2_O 60/40, **2**: 24 min MeOH/H_2_O 70/30, and **3**: 27 min MeOH/H_2_O 80/20 with amounts of 2.6, 1.0 and 1.1 mg, respectively).

Lyophilized sponge sample *Hyrtios* sp. (16 g) was extracted with MeOH (5 × 200 mL, sonicated each time for 15 min) at room temperature. The five extracts were combined, filtered and concentrated under reduced pressure to yield 3 g of MeOH extract which was treated twice through a solvent partition using CH_2_Cl_2_ (150 mL) and MeOH/H_2_O 1:1 (150 mL). After solvent evaporation, 0.9 g of an anti-PLA_2_ active CH_2_Cl_2_ extract and 2.1 g of an anti-PLA_2_ active MeOH/H_2_O were obtained. The MeOH/H_2_O extract was chromatographed on a C18 SPE column (Phenomenex) and eluted with H_2_O, H_2_O/MeOH 2:1, H_2_O/MeOH 1:2 and MeOH (100 mL of each). The anti-PLA_2_ active fraction H_2_O/MeOH 1:2 (220 mg) was eluted from an Sephadex LH 20 column (GE Healthcare) with MeOH to give an anti-PLA_2_ active yellow fraction (30 mg) which was further submitted to semi-preparative reversed-phase HPLC column chromatography (Interchim, Uptisphere C18 7.8 × 250 mm) with increasing amounts of CH_3_CN/0.1% formic acid in H_2_O/0.1% formic acid as eluent (flow rate: 3 mL/min, wavelength: 254 nm) through a linear gradient for 30 min. Five peaks between 12 and 22 min were obtained, and further purified through an analytical reversed-phase HPLC column (Interchim, Uptisphere C18 4.6 × 250 mm) with increasing amounts of CH_3_CN/0.1% formic acid in H_2_O/0.1% formic acid as eluent (flow rate: 1 mL/min, wavelength: 254 nm) through a linear gradient for 30 min and yielded pure compounds **5**–**9** (0.5 mg for **5**, 1.4 mg for **6**, 1.3 mg for **7**, 1.7 mg for **8**, 3.0 mg for **9**). An aliquot of the crude CH_2_Cl_2_ extract (0.4 g) was chromatographed on a silica gel column, using a linear gradient of acetone in CH_2_Cl_2_ as eluent. The 50% acetone fraction afforded pure **4** (4 mg).

(*1*H*-Indol-3-yl*)*oxoacetamide* (**1**). White powder; ESI-MS *m/z* 189.0677 [M + H]^+^ (calcd. 189.0658 for C_10_H_9_N_2_O_2_); spectroscopic data matched those previously published [4].

(*1*H*-Indol-3-yl*)*oxoacetic acid methyl ester* (**2**). Yellow powder; ESI-MS *m/z* 204.0657 [M + H]^+^ (calcd. 204.0660 for C_11_H_10_NO_3_); spectroscopic data matched those previously published [4].

*6-Bromoindole-3-carbaldehyde* (**3**). Yellow needles; ESI-MS *m/z* 223/225, *m/z* 223.9713 [M + H]^+^ (calcd. 223.9711 for C_9_H_7_NO^79^Br); spectroscopic data matched those previously published [5].

*Aureol* (**4**). Brown powder; ESI-MS *m/z* 315.2312 [M + H]^+^ (calcd. 315.2324 for C_21_H_31_O_2_); spectroscopic data matched those previously published [6].

*5,6-Dibromotryptamine* (**5**). Light brown powder; ESI-MS *m/z* 317/319/321, *m/z* 316.9302 [M + H]^+^ (*m* calcd. 316.9289 for C_10_H_11_N_2_^79^Br_2_); spectroscopic data matched those previously published [7].

*N-Methyl-5,6-dibromotryptamine* (**6**). Light brown powder; ESI-MS *m/z* 331/333/335, *m/z* 330.9459 [M + H]^+^ (calcd. 330.9439 for C_11_H_13_N_2_^79^Br_2_); spectroscopic data matched those previously published [7].

*N,N-Dimethyl-5,6-dibromotryptamine* (**7**). Light brown powder; ESI-MS *m/z* 345/347/349, *m/z* 344.9557 [M + H]^+^ (calcd. 344.9602 for C_12_H_15_N_2_^79^Br_2_); spectroscopic data matched those previously published [8].

*5,6-*l-*Dibromoabrine* (**8**). Light brown powder; [α]^20^_D_ +17 (*c* 0.05, MeOH-1 N HCl, 8:2) (lit.[9] +44 (*c* 0.05, 1 N HCl)); ESI-MS *m/z* 375/377/379, *m/z* 374.9361 [M + H]^+^ (calcd. 374.9338 for C_12_H_13_N_2_O_2_^79^Br_2_); spectroscopic data matched those previously published [9].

*5,6-Dibromo-*l*-hypaphorine* (**9**). Pale yellow oil; [*α*]^20^_D_ +28 (*c* 0.06, MeOH–1 N HCl, 8:2); UV (EtOH) λ_max_ (log ε) 210 (11,700), 230 (20,645), 294 (2,903) nm; IR (dry film) ν_max_ 3402, 2924, 1597, 1357 cm^−1^; For ^1^H and ^13^C NMR data, see Table 1; ESI-MS *m/z* 403/405/407, *m/z* 402.9667 [M + H]^+^ (calcd 402.9651 for C_14_H_17_N_2_O_2_^79^Br_2_).

### 3.4. PLA_2_ Inhibition Assay

Bioassay guided fractionation was based on a colorimetric bioassay [14]. Assays were performed in duplicate in 96 well plates and read on a CERES 900 spectrophotometer. Extracts (250 μg) or fractions (100 μg) dissolved in 10 μL of DMSO were incubated with 2 μL of a PLA_2_ solution (1 mg/mL in DMSO) from *Apis mellifera* bee venom (Sigma) for 1 hr at 25 °C. Then 198 μL of the substrate solution l-*α*-lecithin (Sigma) 3.5 mM containing Triton X-100 (7 mM), NaCl (100 mM), CaCl_2_ (10 mM) and red phenol (0.055 mM) as colorimetric indicator, at pH 7.6 were added and the absorbance at 550 nm read at time 0 and 5 min. Percent inhibition of the enzyme activity was determined by comparison with a control without drug. Manoalide (Aldrich) was used as a positive control.

### 3.5. Antioxidant Assays

*Qualitative DPPH screening*: The potential antioxidant activity of crude extracts and pure compounds were screening using the scavenging activity of the DPPH (Sigma) free radicals. Active extracts were visualized by spraying a purple DPPH solution (2 mg/mL in MeOH) on a Tlc plate (Merck, Silica gel 60 F_254_), where compounds have been deposited. Immediate discoloration of DPPH around tested samples reveals their antioxidant activity.

*Quantitative ORAC assay*: The antioxidant activity of pure compounds was assessed with the ORAC assay. The ORAC assay is a kinetic assay measuring the decrease in fluorescence of fluorescein (FL) (Sigma) by adding the oxidative species AAPH (Aldrich, 2.2′-azobis(2-amidinopropane dihydrochloride). Thus, antioxidant protection of compounds was evaluated over time. The antioxidant Trolox (Aldrich, 6-hydroxy-2,5,7,8-tetramethylchroman-2-carboxylic acid) was used as a positive control delaying the loss of FL fluorescence in a dose dependent manner [15,16]. The antioxidant activity is normalized to equivalent Trolox units to quantify the antioxidant activity of each compound. The assay was performed with a spectrofluorimeter Berthold Mithras LB 940. Reaction mixtures containing 25 μL of different 2-fold dilutions of pure compounds (dissolved in phosphate buffer 75 mM, pH 7.4 containing 5% DMSO) or Trolox (200 μM–12.5 μM) and 150 μL of FL solution (10 nM in phosphate buffer) were distributed in 96 well microplates in triplicate and incubated at 37°C for 15 min. Fluorescence was measured (Ex. 485 nm, Em. 520 nm) every 90 sec to determine the background signal. After 3 cycles of measurement, 25 μL of an AAPH solution (240 mM in phosphate buffer) was added via an automated injector and 60 fluorescence measurements were taken over a 90 min time period. The final relative ORAC values for tested compounds were calculated by using a regression equation and were expressed as Trolox equivalents according to Ou *et al*. [16]. Trolox and ascorbic acid (Acros Organics) solutions were used as positive controls and FL solution with AAPH as blank.

### 3.6. Cytotoxicity Assay

The human KB cell line was obtained from ECACC (Salisbury, UK) and grown in D-MEM medium supplemented with 10% fetal calf serum (Invitrogen), in the presence of penicillin, streptomycin and fungizone in a 75 cm^2^ flask under 5% CO_2_. Cells were plated in 96-well tissue culture microplates at a density of 650 cells/well in 200 μL medium and treated 24 hrs later with compounds dissolved in DMSO using a Biomek 3000 automate (Beckman-Coulter). Controls received the same volume of DMSO (1% final volume). After 72 hrs exposure MTS reagent (Celltiter 96 Aqueous One solution, Promega) was added and incubated for 3 hrs at 37°C: the absorbance was monitored at 490 nm and results expressed as the inhibition of cell proliferation calculated as the ratio [(1 – (OD_490_ treated/OD_490_ control)) × 100]. For IC_50_ determinations (50% inhibition of cell proliferation) experiments were performed with compound concentrations ranging from 1 μM to 100 μM in duplicate.

## 4. Conclusions

In conclusion, our search for new inhibitors of PLA_2_ and/or antioxidant natural products has led to the investigation of specimens of the South Pacific marine sponges *Rhopaloeides odorabile* and *Hyrtios* sp. Eight indole derivatives including the new 5,6-dibromo-l-hypaphorine (**9**), and the sesquiterpene aureol (**4**) were isolated and their chemical structures were resolved by spectroscopic analysis. Evaluation of anti-PLA_2_ and antioxidant activities of the series led to the identification of both **4** and **9** as potential antioxidant compounds. In contrast to **4**, the new derivative **9** did not show any cytotoxic activity towards the human KB cancer cell line. Consequently, **9** could be promising in cosmetics and/or in pharmaceutics due to its anti-inflammatory and antioxidant potentials.

## Figures and Tables

**Table 1.** NMR spectroscopic data of 5,6-dibromo-l-hypaphorine (**9**)^a^.

**no.**	**^1^H**	**^13^C**	**HMBC^b^**
1-NH	11.20 brs	-	2, 3
2	7.27 s	126.8	3, 3a, 7a
3		109.5	
3a		128.2	
4	8.02 s	123.1	3, 5, 6, 7a
5		112.6 ^c^	
6		114.8 ^c^	
7	7.72 s	116.0	3a, 5, 6, 7a
7a		135.7	
8	3.21 m	24.2	2, 3, 3a, 9, 10
9	3.67 dd (10.1, 3.3)	78.2	3, 8, 10
10		167.0	
OH	8.45 brs		10
N(CH_3_)_3_	3.17 s	51.0	

a Measured in DMSO-*d*_6_ at 400 MHz for ^1^H and 75.15 MHz for ^13^C, *J* in Hz;

b HMBC correlations optimized for 7 Hz, are from proton(s) stated to the indicated carbon;

c Assignments may be interchanged.

**Table 2.** Biological activities of compounds **1**–**9**.

**Compound**	**PLA_2_^a^**	**ORAC _FL_^b^**
(1*H*-Indol-3-yl)oxoacetamide (**1**)	1.17 ± 0.05	nt
(1*H*-Indol-3-yl)oxoacetic acid methyl ester (**2**)	1.11 ± 0.33	nt
6-Bromoindole-3-carbaldehyde (**3**)	1.27 ± 0.06	nt
Aureol (**4**)	0.46 ± 0.02	0.29 ± 0.03
5,6-Dibromotryptamine (**5**)	0.62 ± 0.01	nt
*N*-Methyl-5,6-dibromotryptamine (**6**)	0.33 ± 0.03	nt
*N*,*N*-Dimethyl-5,6-dibromotryptamine (**7**)	0.77 ± 0.05	0.06 ± 0.01
5,6-Dibromoabrine (**8**)	0.30 ± 0.01	0.07 ± 0.01
5,6-Dibromo-l-hypaphorine (**9**)	0.20 ± 0.01	0.22 ± 0.04

a IC_50_ values (mM ± SEM; n = 2) on bee venom PLA_2_. Manoalide (positive control) IC_50_ 0.5 ± 0.05 μM.

b ORAC values are expressed as relative Trolox equivalent. Fluorescein (FL). Relative ORAC value = [(AUC product – AUC blank)/(AUC Trolox – AUC blank)] × (molarity Trolox/molarity product), (n = 3). Molarity in μM. AUC: Area Under the Curve. AUC blank = AUC obtained for the control FL + AAPH. Ascorbic acid (positive control) ORAC_FL_ 0.95 ± 0.02; nt: not tested.
